# Systematic literature review of risk factors for cervical cancer in the Chinese population

**DOI:** 10.1177/1745506518816599

**Published:** 2018-12-14

**Authors:** Xiao Li, Shang Ying Hu, Yunkun He, Leyla Hernandez Donoso, Kelly Qiao Qu, Georges Van Kriekinge, Fang Hui Zhao

**Affiliations:** 1 Health Economics Department, GSK, Wavre, Belgium; 2 Department of Cancer Epidemiology, Cancer Hospital/Institute, Chinese Academy of Medical Sciences and Peking Union Medical College, Beijing, China; 3 GSK, Shanghai, China; 4 VBU (Vaccine Business Unit), Takeda Pharmaceutical Company Limited, Cambridge, MA, USA; 5 Amaris, Toronto, ON, Canada

**Keywords:** cervical cancer, China, human papillomavirus, risk factors, sexually transmitted infections, uterine cervical neoplasms

## Abstract

**Objectives:**

Human papillomavirus is the necessary cause of cervical cancer, in particular the human papillomavirus-16/18 strains, which have been detected in ~70% of all cervical cancer cases worldwide. This study aims to assess whether other cofactors, which might be specific for the Chinese population, are involved in the development of cervical cancer. These findings may support the future direction of cervical cancer prevention.

**Study Design:**

Systematic literature review.

**Methods:**

The following databases were searched: MEDLINE, MEDLINE-IN-PROCESS, EMBASE, China National Knowledge Infrastructure, Wanfang Data and Chongqing VIP Information. The target population were adolescents or adults from mainland China. All observational studies irrespective of intervention or comparator reporting risk factors for cervical cancer were included. The Newcastle-Ottawa Scale was used to assess study quality. The impact of each outcome was reported in numerical terms.

**Results:**

A total of 2,676 articles were screened. A total of 21 articles met the inclusion criteria. All studies were case-controlled designs mostly conducted in hospitals of South-Eastern China. A total of 18 studies reported lifestyle behaviours as significant influencing factors in the development of cervical cancer. Sexual behaviour, gestational factors, screening history, disease history and socio-demographics status were reported as significant risk factors for cervical cancer development.

**Conclusion:**

This review provides an up-to-date insight of current cervical cancer risk factors in China. Due to the heterogeneity of the results, further evaluation is recommended to determine the association of these risk factors to the overall risk of cervical cancer.

## Introduction

Human papillomavirus (HPV) refers to a family of viruses that infect epithelial tissues, such as the skin, cervix, vulva, vagina, anus, mouth and throat,^1–3^ primarily transmitted through sexual contact.^4,5^ A total of 66 HPV types specifically infect the genital mucosae. Numerous strains are associated with an increased risk of cervical cancer (CC) development, including HPV-16 and HPV-18 found most frequently in CC specimens.^6,7^ It has been reported that the odds ratio (OR) of developing squamous cell carcinoma from HPV-16 and adenocarcinoma from HPV-18 range from 100 to 900 when compared with individuals with no detectable infection.^8^ HPV has been causally linked to CC^9^ and is estimated to cause up to 70% of all vaginal cancers and 43% of all vulvar cancers.^9^

In China, oncogenic HPV infection in women has been reported as 5%–20%, depending on location and age.^10–14^ The estimated number of new CC cases was reported as 98,900 in 2015.^15^ Annual new CC cases are estimated to increase to 42,000–187,000 by 2050.^15,16^ The reported age-standardised mortality rate (per Chinese standard population) of CC in China was 2.41 in urban areas and 2.87 in rural areas per 100,000 population in 2013.^17^ It was estimated that 30,500 CC deaths would have occurred in 2015.^15^

Risk factors associated with HPV infection were reported in a cross-sectional study performed in local obstetrics and gynaecology clinics. It was reported that age and lifetime number of sexual partners were significant risk factors (p < 0.05) in this population.^11^ However, this study was limited as it did not have a control population for comparison and included a potentially biased study population. Thus, direct association of these risk factors must be determined with more suitable methodologies.

Systematic literature reviews on the association of CC risk factors in China have mostly been published in Chinese language. To widen the scope and summarise all risk factors for CC, this study aimed to conduct an up-to-date systematic review in both English and Chinese databases in English and Chinese language. A better understanding of the epidemiology of CC risk factors in China may provide insight for shaping future CC prevention programmes in the country.

## Methods

### Data source and literature search

**Figure 1.**
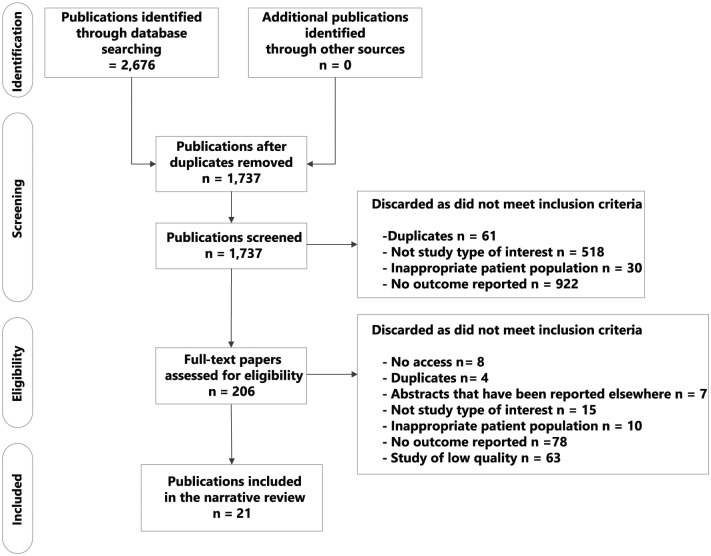
PRISMA diagram showing the selection process of identifying eligible studies.

The present systematic review was reported in accordance with the PRISMA (Preferred Reporting Items for Systematic Reviews and Meta-Analyses) statement.^18^ Adhering to PRISMA guidelines (see Supplementary Table 1), the systematic literature search was conducted on 27 February 2014 on databases MEDLINE, MEDLINE-IN-PROCESS and EMBASE in English, as well as on databases China National Knowledge Infrastructure (CNKI), Wanfang Data and Chongqing VIP Information (CQVIP) in Chinese on 4 March 2014. The search terms were developed using a combination of the following keywords: ‘cervical neoplasm’, ‘cervical cancer’, ‘risk factors’, ‘China’ and ‘Chinese’, without restriction on time span (see Supplementary Table 2). The PRISMA flow diagram is presented in Figure 1.

### Study selection and inclusion/exclusion criteria

All retrieved citations were screened by one reviewer and quality-checked by another using the inclusion/exclusion criteria described below. The title and abstract (where available) of each record were screened based on (1) observational study design, (2) data from sample sizes of more than 20 patients, (3) reported risk factors for CC with numerical values and (4) studies of fair and good quality as defined by the Newcastle-Ottawa Scale (NOS).^19^ Reviews, case series, case reports, clinical trials, animal, in vitro or in vivo studies were excluded. Other exclusion criteria included (1) duplicated studies, (2) abstracts reported elsewhere, (3) non-relevant study type, (4) inappropriate patient population and (5) no reported outcome. Eligible studies were included for full-text review.

### Data extraction and synthesis

Two independent reviewers extracted relevant information from the included studies for review. Data extraction was conducted in the language of the original publication and translated into English for analysis. In the extraction sheet, ORs from multivariate analyses, regardless of statistical significance, were recorded. When unavailable, ORs from univariate analysis were extracted. A narrative report was generated to document the results of the analyses with descriptive summaries. The evidence synthesised was stratified by different categories of risk factors.

### Quality assessment

Quality assessment was performed independently by two reviewers, using NOS (see Supplementary Table 3).^19^ The NOS assesses the quality of a study based on three parameters: (1) selection of population (maximum score of 4 points), (2) comparability of the groups (maximum score of 2 points) and (3) ascertainment of the exposure (maximum score of 3 points).^19^ Studies were selected in terms of quality, based on the score obtained for each of these parameters. Only those with fair (4–6 points) and good (7–9 points) scores were included for analysis. Any disagreements were settled by discussion with a third reviewer.

## Results

### Identification of studies

A total of 2,676 eligible publications were identified. After 939 duplicates were removed, 1,737 studies remained. The screening of titles and abstracts led to 206 full-text reviews. After reviewing the full texts, 21 publications were considered for narrative review (Figure 1).

### Study characteristics

All selected publications were case–control studies, based on a one-to-one matching, published between 1986 and 2014. The majority of publications reported findings from provinces located in South-Eastern China. Data were collected from 1973 to 2013 from patients aged 18–85 years. The mean sample size was 100 for cases and 150 for controls. A total of 13 studies employed multivariate regression analysis to estimate the effect of the investigated factors on the risk for CC.

Most studies explored the general lifestyle and socio-demographic risk factors for CC (Table 1). Four studies focused on the risks associated with screening attendance, tubal ligation and use of intrauterine devices, diet and supplements, passive smoking and tea-drinking.^20–23^

**Table 1.** Study characteristics of all the included case–control studies.

Study reference	Study period (years)	Age of patients (years)	Population sample size	
			Case	Control
s.n.^24^	1974–1975, 1978–1979		306	306
Zhang et al.^20^	1973–1975	35–85	119	545
Zhang et al.^20^	1974–1985	35–85	119	545
Zhang and Xu^25^	October 1987–November 1988		125	125
Peng et al.^26^	June 1987–November 1988	Case mean = 53.7, SD = 10.4 Controls mean = 51.7, SD = 10.8	101	146
Wang et al.^27^	>1980	Mean = 62.4	100	100
Dong et al.^28^	August 1995–September 1996	25–70 (mean = 47)	43	327
Li et al.^21^	January 1989–May 1991	30–77	272	893
Cai et al.^29^	2003–2004	18+	110	110
Wang et al.^30^	2001–2002	Case = 24–78 (median: 51,39) Control = 25–75 (median: 51,43)	129	143
Ma et al.^22^	January–May 2004	Case = 50.56, SD = 9.61 Control = 46.24, SD = 9.39	133	133
Kan et al.^31^	September 2006–July 2008	Case = 21–85 (median: 46) Control = 20–78 (median: 44.5)	893	1786
Zhang et al.^32^	June–December 2004	20–78 (median: 43.2)	286	858
Li et al.^33^	January 2007–December 2009		80	80
Li et al.^34^	September 2007–June 2010	Case = 28–60 (mean = 43.16, SD = 6.8) Control = 29–58 (mean = 42.19, SD = 5.2)	112	200
Zeng^35^	2010	20–55	129	200
Liu et al.^36^	March 2009–May 2012	Case = 26–68 (median: 42.7) Control = 35–66 (median: 43.1)	183	366
Jiang^37^	2011–2013	Case = 43–65, mean = 55.3, SD = 7.9 Control = 39–71, mean = 52.6, SD = 6.5	42	45
Gao et al.^38^	August 2009–July 2010	Case = 25–65, mean = 44.52; Control = 24–59, mean = 44.13	100	799
Wang and Zhou^39^	January 2009–April 2013	Case = 24–51, mean = 40.5; Control = 25–49, mean = 39.1	80	80
Nie et al.^23^	July 2007–December 2008	Case = 24–62, mean = 42.9, SD = 8.009	165	248

SD: standard deviation; s.n.: sine nomine.

### Methodological quality of the studies

The NOS assessment scale rated the quality of the publications based on selection, comparability and exposure. Point and quality allocation are reported in Table 2. For comparability, the ORs were adjusted for at least one important factor (age, sex, location, population size). Exposure was ascertained in most cases by secure clinical records (n = 17), structured interviews (n = 2) or both (n = 2). The same method of ascertainment was used for both cases and controls. The overall quality of the studies qualified as fair (n = 9, 43%) or good (n = 12, 57%) (Table 2).

**Table 2.** Quality assessment of the 21 included studies by Newcastle-Ottawa Scale.

Study	Detailed scoring								Domain score			Quality
	Case definition	Representativeness of cases	Selection of controls	Definition of controls	Comparability	Ascertainment of exposure	Same method for case and controls for ascertainment	Non-response rate	Selection domain	Comparability domain	Outcome domain	
s.n.^24^	1	1	1	1	2	2	1	0	4	2	3	Good
Zhang et al.^20^	1	1	1	1	2	2	1	0	4	2	3	Good
Zhang et al.^20^	1	1	1	1	2	2	1	0	4	2	3	Good
Zhang and Xu^25^	1	1	0	1	2	2	1	0	3	2	3	Good
Peng et al.^26^	1	1	0	1	2	2	1	0	3	2	3	Good
Dong et al.^28^	1	1	1	1	2	2	1	0	4	2	3	Good
Li et al.^21^	1	1	0	1	2	2	1	0	3	2	3	Good
Cai et al.^29^	1	1	0	1	2	2	1	0	3	2	3	Good
Wang et al.^27^	0	1	0	1	1	1	1	0	2	1	2	Fair
Wang et al.^30^	0	1	0	1	1	1	1	0	2	1	2	Fair
Ma et al.^22^	0	1	0	1	1	1	1	0	2	1	2	Fair
Kan et al.^31^	0	1	0	1	1	2	1	0	2	1	3	Fair
Zhang et al.^32^	1	1	1	1	2	1	1	0	4	2	2	Good
Li et al.^33^	0	1	0	1	1	1	1	0	2	1	2	Fair
Li et al.^34^	0	1	0	1	1	1	1	0	2	1	2	Fair
Zeng^35^	1	1	0	0	1	1	1	0	2	1	2	Fair
Liu et al.^36^	1	1	0	1	2	2	1	0	3	2	3	Good
Jiang^37^	0	1	0	1	1	1	1	0	2	1	2	Fair
Gao et al.^38^	1	1	0	0	1	1	1	0	2	1	2	Fair
Wang and Zhou^39^	1	1	0	1	2	1	1	0	3	2	2	Good
Nie et al.^23^	1	1	1	1	2	2	1	0	4	2	3	Good

s.n.: sine nomine.

### Risk factors associated with CC

#### Overview of the risk factors

Six risk factor categories were identified in this review: socio-demographics, lifestyle, sexual behaviour and marriage, gestational factors, CC screening, gynaecological diseases, and other factors. Geographical distribution and the corresponding risk factors for each study location are presented in Supplementary Table 4.

Lifestyle behaviour was the most frequently assessed risk factor (considered in 18 studies; 86%), while socio-demographic factors were included in the least number of studies (6 studies; 29%). The specific risk factors and their frequency are assessed in each sub-category and are presented in Supplementary Table 5. The number of risk factors reported in different categorical ranges of OR is reported in Supplementary Table 6.

#### Risk factor 1: socio-demographics

Three types of socio-demographic risk factors were presented in the studies: (1) education level (i.e. primary school, secondary school, high school/college/university), (2) economic status (i.e. low or high income) and (3) occupation (i.e. manual labour, intellectual job, housewife).

Lü et al.^40^ reported a statistical significant effect of higher income as protective factor for CC after controlling for confounders in the multivariate analysis (OR = 0.5). One study conducted by Li et al.^33^ identified an association between limited education and the risk for developing CC (OR: 1.06) based on a multivariate analysis. Other studies also indicated that higher education (above high school) and occupation (housewife; intellectual job) are protective factors (OR <1) against CC.^31,33^ However, this cannot be concluded as true associations due to the lack of multivariate analysis and significance reporting. In a univariate analysis from Zhang and Xu,^25^ lower economic status indicated a more than twofold increase in risk for CC.

#### Risk factor 2: lifestyle

Addictions (i.e. smoking), personal hygiene and dietary habits were analysed and found to be associated with CC in China.

Both active and passive smoking was associated with a significant CC risk increase in several analyses (range of ORs: 1.844–4.88).^21,23,28,31–37^ Poor personal hygiene was also associated with a higher risk for CC.^30,33,41^ In a multivariate analysis controlled for confounders, Zeng^35^ reported good personal hygiene as a protective factor against CC (OR = 0.273).

Five articles investigated the association between dietary habits and tea consumption and risk for CC. Two multivariate analyses reported a statistically significant effect of tea-intake in preventing CC development in approximately 20%–50% of the sampled population.^23,34^ Three studies found that dietary habits could affect the risk of CC positively or negatively depending on the specific diet of the individual.^22,26,35^ However, as ingredients were not specified, conclusions on diet and CC risk could not be drawn.

#### Risk factor 3: sexual behaviour and marital status

Factors relating to sexual behaviour were analysed and the association of these factors with CC risk are age at first sexual debut, age at marriage, number of sexual pattern and others.

Four studies, which controlled for confounders, reported a significantly increased risk for CC for early sexual debut age 18–20 years.^29,30,34,36^ Two studies further concluded that marriage at or above 20 years old was significantly associated with a decreased risk for CC.^23,42^ Zhang et al.^32^ reported an OR 1.36 of CC risk from marriage in a multivariate analysis. Only one study showed no association between age of first marriage and CC risk.^21^

Several studies analysed the relationship between the lifetime number of sexual partners and CC risk. One study reported an OR of 7.089 for CC in individuals who had more than one sexual partner compared with those with only one.^35^ An increased number of sexual partners of the spouse was also associated with an increased CC risk.^20,24^

#### Risk factor 4: gestational risk factors

Gestational risk factors included contraception, number of pregnancies and live births, delivery history, age of first pregnancy and age of first delivery, menarche, menopause and menstruation period.

Three multivariate studies analysed the risk of contraceptive use and their link with CC, showing that oral contraceptive users have a more than two times higher risk for CC (OR: 2.419).^35,36,43^ Condoms and other contraceptives demonstrated a 40% lower CC risk (OR: 0.44). These data were consistent with previously reported data by Li et al.;^33^ however, they were not found to be statistically significant.

In two multivariate analyses, a higher pregnancy rate was significantly associated with increased CC risk (OR = 4.2 and 3.8, respectively), while higher number of live births increased the risk for CC.^29,30,33,39^ Similar findings were reported by Cai et al.,^29^ who reported a significant OR of 16.8 (p < 0.05) for CC in women with more than three live births.

Menopause and early menarche (<14 years old) were significantly associated with an increased risk for CC.^35,39^ Zeng^35^ showed that early menarche is protective of CC with a low OR of 0.279. However, Wang et al.^30^ showed an increased risk of CC with early menarche (OR = 3.242), as well as a significantly decreased risk for CC for women who had had menopause (OR = 0.68). Three studies concluded that abortion significantly increased the risk for CC in the studied populations (ORs = 2.45, 3.91 and 6.11, respectively).^35,36,39^

#### Risk factor 5: CC screening and gynaecological diseases

In total, 14 articles reported on the potential association of CC screening and presence of disease as risk factors for CC.^24,25,27,29–33,35–39,41^ These included HPV infection, cervical screening history, sexually transmitted diseases (STDs) and gynaecological disorders.

All studies reported a strong association between HPV infection and CC development. Cai et al.^29^ found that prolonged delays between Pap smears increased the risk for CC due to HPV infection. Two articles found that STDs had a significant impact on CC risk, but required further verification.^37,43^

Gynaecological disorders/diseases were most associated with CC risk. Cervical diseases, specifically cervical erosion, were identified as a significant risk for CC by all articles but one.^20^ Disorders such as pelvic and cervical inflammation increased the risk of CC at least twofold (OR = 2.377 and 5.496, respectively).^37,39^ Genetic familial diseases have also been indicated as a CC risk factor.^36,43^ All studies, apart from Cai et al.,^29^ failed to associate a significant correlation between history of CC screening and CC risk.

#### Risk factor 6: other factors

Other risk factors included disease knowledge, mental disease, hospital visits, foreskin status and penile disease of the husband, biomarker levels and bodily measures.

Three studies reported significant, positive correlations between foreskin status and CC risk.^36^ Moreover, two studies reported that the penile disease or cancer in the spouse significantly increased CC risk.^31,36^

Gao et al.^38^ demonstrated that diastolic blood pressure higher than 90 mmHg significantly reduced the risk of CC by 10 (OR = 0.12). A study by Li et al.^34^ showed that folic acid levels above 15 µg/dL reduced the risk for CC by 61% (OR = 0.389; p < 0.05).

Three NOS fair-quality studies reported mental illness as a significant risk factor for CC, with one study showing that mental health status and stress increased the risk for CC by four.^20,43^ However, one study with a NOS good-quality score did not find a significant association between mental health and CC risk.^26^ Other factors that have been linked to lowering the risk for CC are listed in Supplementary Table 5.

## Discussion

Results from this systematic literature review summarised the risk factors for CC in China. Lifestyle (smoking status and hygiene), sexual behaviour (non-circumcision), gestational factors (contraception, number of pregnancies, abortion or late menopause age), cervical screening (abnormal Pap smears or longer durations between screenings) and gynaecological disorder/disease all increased the risk of CC. This review also identified several protective factors against CC. This included higher income, good hygiene, green tea-intake, use of condoms and higher levels of folic acid.

The majority of studies reported on populations in South-Eastern China and the patient population included adult or adolescent Chinese women in mainland China from provincial or local hospitals. The exposure statuses to risk factors were collected through a mix of questionnaires and medical files. A large heterogeneity was noted in terms of defining risk factor categories, analytical methods, quality of the studies and geographical location, which enabled diverse socio-demographic results.

All the studies included in this review are case–control studies. More cohort studies and cross-sectional studies in the CC risk factor research are also urgently needed as the understanding of the CC risk factors shows several gaps. Moreover, in the majority of the case–control studies, the authors neither disclose the questionnaires nor report how the questionnaires were designed. Therefore, a long list of risk factors was identified in this literature review (Supplemental Table 5) and they were summarised under six categories in the main body of this review. A standardised questionnaire with limited number of risk factors would help to focus the research, reduce the heterogeneity and improve the comparison across geographical regions. Furthermore, great variability was noted in terms of how the risk factors were considered in statistical analyses (i.e. univariate analysis vs multivariate analysis), which makes the interpretation and comparison of the findings challenging.

The results from this review coincide with two meta-analyses determining CC risk factors in Chinese women and published in Chinese language.^40,44^ There were several similarities between these reviews, including the number of articles retrieved, protective factors and risk factors for CC. Risk factors that reached statistical significance reported by Zhang et al.^44^ included educational background (⩽9 years; p < 0.001), income, occupation, age of sexual debut (⩽20 years; p < 0.001), first pregnancy (⩽21 years; p < 0.001), marriage and menopause, HPV infection, multiple marriages (⩾2, p < 0.001) or births (⩾3, p < 0.001), two or more sexual partners, history of malignancy and smoking (p < 0.001). These findings were similar to findings reported by Kan et al.^31^

The main strength of this review is the substantial number of both English- and Chinese-language databases that were searched and analysed following the gold standard of systematic review, including disclosure of the detailed search query, selection criteria and quality assessment. It provides a comprehensive coverage of available knowledge on this topic, covering scientific research over a period of 28 years. Although the presence of risk and pattern of risk-taking may have changed over such a long period of time, the focus of scientific research on CC risk may have (and most likely has) changed over time in mainland China. Some risk factors have been reported consistently, such as sexual behaviour and marital status, CC screening and gynaecological disease. HPV infections were reported more frequently in those studies dating from the publication year 2008 onwards with better access to laboratory testing. Other risk factors may no longer be captured in more recent research (after year 2000), such as risk factors regarding personal hygiene or sanitary napkins use and genital washing. In the Supplementary Table 5, all the risk factors and year of the study are presented providing an overview of research changes throughout these 28 years.

Limitations of this study include the potential for bias, variability of quality and limited access to all relevant data and study design since the review focused solely on published papers. Selection bias may have occurred as only one reviewer selected the studies in this review. However, we believe that the research protocol was methodologically strong to ensure a reliable study selection. The main limitation lies in how quality was assessed, which significantly varied between studies. This may have led to key information not being provided, skewing the NOS scores assigned in certain domains. A large time frame may also increase the complexity of future statistical analyses due to increased heterogeneity.

Further analysis with a formal statistical method, such as meta-regression, could provide a clearer picture of what is currently known and the estimated impact of each identified risk factor. Involving local clinical experts could provide insights into the design of the statistical analysis plan, scrutinising each risk factor and deciding which factors should be included. The inclusion of certain risk factors could be debated and considered when performing further statistical analysis, based on the strength of association, adjustments for confounders and the biological plausibility of the CC risk factors addressed in this review.

An expert review held in Beijing in 2016 recommended verifying articles against journals published in a ‘core journal’ list. Three lists exist in China: Peking University Chinese Core Journal List, China Science Citation Database and Chinese Social Sciences Citation Index. Limiting article selection using core journals could be used as a quality assessment tool in combination with NOS in Chinese-language articles.

## Conclusion

This review provided an up-to-date insight of the risk factors for CC in China. The main findings of this review are that other factors in conjunction with HPV infection can contribute to CC development, including socio-demographic status, age at sexual debut, number of sexual partners, pregnancies or deliveries, mental health status and penile condition of the husband and cigarette smoking. Further evaluation is needed to equate the association between these risk factors and overall CC risk.

## Supplemental Material

Revised_Version_Suppl_Mat_CC_Risk_factors_in_China_Clean_corrected_JxG – Supplemental material for Systematic literature review of risk factors for cervical cancer in the Chinese population Click here for additional data file. Supplemental material, Revised_Version_Suppl_Mat_CC_Risk_factors_in_China_Clean_corrected_JxG for Systematic literature review of risk factors for cervical cancer in the Chinese population by Xiao Li, Shang Ying Hu, Yunkun He, Leyla Hernandez Donoso, Kelly Qiao Qu, Georges Van Kriekinge and Fang Hui Zhao in Women’s Health
