# Supplementary material for: Systematic literature review of risk factors for cervical cancer in the Chinese population
Source: Womens Health (Lond). 2018 Dec 14;14:1745506518816599. doi: 10.1177/1745506518816599 (PMC6300867; doi:10.1177/1745506518816599)
Supplement: Revised_Version_Suppl_Mat_CC_Risk_factors_in_China_Clean_corrected_JxG – Supplemental material for Systematic literature review of risk factors for cervical cancer in the Chinese population [file Revised_Version_Suppl_Mat_CC_Risk_factors_in_China_Clean_corrected_JxG.pdf]

Systematic literature review of risk factors for cervical cancer in the Chinese population

Supplementary Material

Xiao Li, Shang Ying Hu, Yunkun He, Leyla Hernandez Donoso, Kelly Qiao Qu, Georges Van Kriekinge, Fang Hui Zhao

Supplementary Table 1: PRISMA checklist 3
Supplementary Table 2: Search strategies 4
Supplementary Table 3: Newcastle-Ottawa quality assessment for case-control studies 5
Supplementary Table 4: Geographical overview of risk factors identified for included studies 7
Supplementary Table 5: Risk factor analysis 8
Supplementary Table 6: Counts of risk factors under different categorical ranges of odds ratios 20
References 23

Section/topic	#	Checklist item	Reported on page #
**TITLE**			
Title	1	Identify the report as a systematic review, meta-analysis, or both.	1 (title page)
**ABSTRACT**			
Structured summary	2	Provide a structured summary including, as applicable: background; objectives; data sources; study eligibility criteria, participants, and interventions; study appraisal and synthesis methods; results; limitations; conclusions and implications of key findings; systematic review registration number.	2 (Abstract)
**INTRODUCTION**			
Rationale	3	Describe the rationale for the review in the context of what is already known.	3
Objectives	4	Provide an explicit statement of questions being addressed with reference to participants, interventions, comparisons, outcomes, and study design (PICOS).	3
**METHODS**			
Protocol and registration	5	Indicate if a review protocol exists, if and where it can be accessed (e.g., Web address), and, if available, provide registration information including registration number.	N/A
Eligibility criteria	6	Specify study characteristics (e.g., PICOS, length of follow-up) and report characteristics (e.g., years considered, language, publication status) used as criteria for eligibility, giving rationale.	4
Information sources	7	Describe all information sources (e.g., databases with dates of coverage, contact with study authors to identify additional studies) in the search and date last searched.	4
Search	8	Present full electronic search strategy for at least one database, including any limits used, such that it could be repeated.	Supplementary Table 1
Study selection	9	State the process for selecting studies (i.e., screening, eligibility, included in systematic review, and, if applicable, included in the meta-analysis).	4
Data collection process	10	Describe method of data extraction from reports (e.g., piloted forms, independently, in duplicate) and any processes for obtaining and confirming data from investigators.	4
Data items	11	List and define all variables for which data were sought (e.g., PICOS, funding sources) and any assumptions and simplifications made.	N/A
Risk of bias in individual studies	12	Describe methods used for assessing risk of bias of individual studies (including specification of whether this was done at the study or outcome level), and how this information is to be used in any data synthesis.	N/A
Summary measures	13	State the principal summary measures (e.g., risk ratio, difference in means).	4

Synthesis of results	14	Describe the methods of handling data and combining results of studies, if done, including measures of consistency (e.g., I^2) for each meta-analysis.	4

Supplementary Table 1: PRISMA checklist

Section/topic	#	Checklist item	Reported on page #
Risk of bias across studies	15	Specify any assessment of risk of bias that may affect the cumulative evidence (e.g., publication bias, selective reporting within studies).	N/A
Additional analyses	16	Describe methods of additional analyses (e.g., sensitivity or subgroup analyses, meta-regression), if done, indicating which were pre-specified.	4
**RESULTS**			
Study selection	17	Give numbers of studies screened, assessed for eligibility, and included in the review, with reasons for exclusions at each stage, ideally with a flow diagram.	5
Study characteristics	18	For each study, present characteristics for which data were extracted (e.g., study size, PICOS, follow-up period) and provide the citations.	5-9
Risk of bias within studies	19	Present data on risk of bias of each study and, if available, any outcome level assessment (see item 12).	N/A
Results of individual studies	20	For all outcomes considered (benefits or harms), present, for each study: (a) simple summary data for each intervention group (b) effect estimates and confidence intervals, ideally with a forest plot.	N/A
Synthesis of results	21	Present results of each meta-analysis done, including confidence intervals and measures of consistency.	5-9
Risk of bias across studies	22	Present results of any assessment of risk of bias across studies (see Item 15).	N/A
Additional analysis	23	Give results of additional analyses, if done (e.g., sensitivity or subgroup analyses, meta-regression [see Item 16]).	N/A
**DISCUSSION**			
Summary of evidence	24	Summarize the main findings including the strength of evidence for each main outcome; consider their relevance to key groups (e.g., healthcare providers, users, and policy makers).	9-10
Limitations	25	Discuss limitations at study and outcome level (e.g., risk of bias), and at review-level (e.g., incomplete retrieval of identified research, reporting bias).	10
Conclusions	26	Provide a general interpretation of the results in the context of other evidence, and implications for future research.	10
**FUNDING**			
Funding	27	Describe sources of funding for the systematic review and other support (e.g., supply of data); role of funders for the systematic review.	12

From: Moher D, Liberati A, Tetzlaff J, Altman DG, The PRISMA Group (2009). Preferred Reporting Items for Systematic Reviews and Meta-Analyses: The PRISMA Statement. PLoS Med 6(6): e1000097. doi:10.1371/journal.pmed1000097

For more information, visit: **www.prisma-statement.org**.

Supplementary Table 2: Search strategies

(A) MEDLINE and MEDLINE-IN-PROCESS via Ovid
Searched performed on 27th February 2014

#	Search terms	Hits
1	EXP "Uterine Cervical Neoplasms"/	57,561
2	((cervical OR cervix) ADJ3 (neoplasm$1 OR cancer$1)). TI, AB.	33,395
3	OR/1-2	66,788
4	EXP "Risk Factors"/	543,931
5	EXP Algorithms/	167,163
6	((risk ADJ1 factor$1) OR predictive OR predictivit$3 OR prediction$1 OR algorithm$1 OR epidemiol* OR determinant$1). TI, AB.	1,125,561
7	OR/4-6	1,539,210
8	EXP China/	89,669
9	(China OR Chinese). TI, AB.	163,558
10	OR/8-9	193,741
11	3 AND 7 AND 10	259

(B) EMBASE via embase.com
Search performed on 27th February 2014

#	Search terms	Hits
1	'Uterine Cervix Cancer'/EXP	61,556
2	((cervical OR cervix) NEAR/3 (neoplasm OR neoplasms OR cancer OR cancers)): TI, AB	41,614
3	#1 OR #2	74,099
4	'Risk Factor'/EXP	588,403
5	Algorithm/EXP	161,528
6	((risk NEAR/1 (factor OR factors)) OR predictive OR predictivity OR predictivities OR prediction OR predictions OR algorithm OR algorithms OR epidemiology OR epidemiologies OR epidemiologic OR epidemiological OR determinant OR determinants): TI, AB	1,384,405
7	#4 OR #5 OR #6	1,713,381
8	China/EXP	93,936
9	Chinese/EXP	29,746
10	(China OR Chinese): TI, AB	209,891
11	#8 OR #9 OR #10	241,362
12	#3 AND #7 AND #11	252

(C) CNKI
Search performed on 4th March 2014

#	Search terms	Hits
1	(SU='宫颈癌' OR SU='子宫颈癌') AND (KY='危险度' OR KY='危险因素' OR KY='危险性因素' OR KY='风险因素' OR KY='相关因素' OR KY='保护因素' OR KY='影响因素'OR KY='高危因素' OR KY='协同因素' OR KY='预期' OR KY='预测' OR TI='危险度' OR TI='危险因素' OR TI='危险性因素' OR TI='风险因素' OR TI='相关因素' OR TI='保护因素' OR TI='影响因素' OR TI='高危	1,081

	因素' OR TI='协同因素' OR TI='预期' OR TI='预测') NOT (TI='预后' OR TI='复发' OR TI='转移')	

(D) <u>Wanfang Data</u>
Search performed on 4th March 2014

#	**Search terms**	**Hits**
1	(主题:(宫颈癌+子宫颈癌) *题名或关键词:(危险度+危险因素+危险性因素+风险因素+相关因素+保护因素+影响因素+高危因素+协同因素+预期+预测))^题名:(预后+复发+转移)	510

(E) <u>CQVIP</u>
Search performed on 4th March 2014

#	**Search terms**	**Hits**
1	((T=(宫颈癌+子宫颈癌) +R=(宫颈癌+子宫颈癌))*(K=(危险度+危险因素+危险性因素+风险因素+相关因素+保护因素+影响因素+高危因素+协同因素+预期+预测)+T=(危险度+危险因素+危险性因素+风险因素+相关因素+保护因素+影响因素+高危因素+协同因素+预期+预测)))-T=(预后+复发+转移)	574

CQVIP, Wanfang Data and Chongqing VIP Information; CNKI, China National Knowledge Infrastructure

Supplementary Table 3: Newcastle-Ottawa quality assessment for case-control studies

Selection	
1) <u>Is the case definition adequate?</u>	
	a) Yes, with independent validation*
	b) Yes, e.g. record linkage or based on self-reports
	c) No description
2) <u>Representativeness of the cases</u>	
	a) Consecutive or obviously representative series of cases*
	b) Potential for selection biases or not stated
3) <u>Selection of Controls</u>	
	a) Community controls*
	b) Hospital controls
	c) No description
4) <u>Definition of controls</u>	
	a) No history of disease (endpoint)*

	b) No description of source
Comparability	
1) <u>Comparability of cases and controls on the basis of the design or analysis</u>	
	a) Study controls for (select the most important factor)*
	b) Study controls for any additional factor* (this criterion could be modified to indicate specific control for a second important factor)
Exposure	
1) <u>Ascertainment of exposure</u>	a) Secure record*
	b) Structured interview where blind to case/control status*
	c) Interview not blinded to case/control status
	d) Written self-report or medical record only
	e) No description
2) <u>Same method of ascertainment for cases and controls</u>	
	a) Yes*
	b) No
3) <u>Non-response rate</u>	
	a) Same rate for both groups*
	b) Non-respondents described
	c) Rate different and no designation

A study can be awarded a maximum of 1 point for each item within the Selection and Exposure categories. A maximum of 2 points can be given for Comparability.

Supplementary Table 4: Geographical overview of risk factors identified for included studies

Study Reference	Municipality level	Provincial level	Prefecture level	Hospital level	Risk factor identified
s.n. (1986) [1]	NR	Jiangxi	Yichun	NR	Lifestyle, Gestational, Other factors<br>Screening, Sexual behavioural
Zhang et al. (1989) [2]	NR	Jiangxi	NR	NR	Lifestyle, Sexual behavioural
Zhang et al. (1989) [3]	NR	Jiangxi	NR	NR	Lifestyle, Screening
Zhang et al. (1990) [4]	NR	Shandong	NR	Shandong Medical University Hospital, Tumour Hospital and Qian Foshan Hospital	Socio-demographics, Lifestyle, Other factors, Sexual behavioural, Screening
Peng et al. (1991) [5]	NR	Sichuan	NR	The gynaecological oncology clinic of West China University Hospital	Lifestyle
Dong et al. (1998) [6]	NR	Liaoning	NR		Lifestyle, Gestational
Li et al. (2000) [7]	NR	Shandong	Jinan	Tumour Hospital	Lifestyle, Gestational
Cai et al. (2008) [8]	NR	Hubei		Zhongnan Hospital	Screening, gestational
Wang et al.(1992) [9]	NR	Guangdong	Guangzhou	Tumour Hospital attached to Zhongshan Medical University Hospital	Screening, Sexual behaviour<br>Gestational
Wang et al. (2004) [10]	NR	Shanxi	NR	Shanxi Tumour Hospital	Socio-demographics, Lifestyle<br>Gestational, Screening<br>Sexual behaviour
Ma et al. (2005) [11]	NR	Shanxi	NR	Shanxi Gynaecology clinic of Tumour Hospital	Lifestyle, Other factors
Kan et al. (2009) [12]	NR	Shandong	NR	Shandong University's Qilu Hospital, Second Hospital and Tumour Hospital	Socio-demographics, Lifestyle, Other factors, Screening, Sexual behaviour<br>Gestational
Zhang et al. (2010) [13]	Beijing	NR	NR		Lifestyle, Gestational, Screening<br>Sexual behaviour
Li et al. (2010) [14]	NR	Sichuan	Liangshan	Liangshan First People's Hospital	Socio-demographics, Lifestyle<br>Gestational, Other factors, Screening
Li et al. (2011) [15]	NR	Fujian	Xiamen		Lifestyle, Other factors, gestational
Zeng (2012) [16]	NR	Guangdong	Guangzhou	People's Hospital in Dashi, Panyu	Socio-demographics, Lifestyle, Gestational, Other factors, Sexual behaviour, Screening
Liu et al. (2013) [17]	NR	Gansu	Longnan	(1) Longnan People's Hospital;<br>(2) Wudu People's Hospital;<br>(3) Cheng county's People's Hospital	Lifestyle, Gestational, Sexual behaviour, Screening, Other factors

Jiang (2013) [18]	NR	Guangxi	Wuzhou	Wuzhou Red Cross Hospital	Screening, Lifestyle
Gao et al. (2013) [19]	NR	Liaoning	Shenyang	Shenyang Tumour Hospital	Socio-demographics, Gestational<br>Other factors, Screening, Sexual behaviour
Wang and Zhou (2014) [20]	NR	Hunan	Zhuzhou	Zhuzhou Women's and Children's Hospital	Gestational, screening<br>Sexual behaviour
Nie et al. (2014) [21]	NR	Fujian	Xiamen		Lifestyle, Gestational<br>Sexual behaviour, Other factors

NR, not reported; s.n., sine nomine.

Supplementary Table 5: Risk factor analysis

(A) Socio-demographic

Study Reference	Risk factor	Result	Significance	Statistics	Notes
**1. Education**					
Kan et al. (2009) [12]	Education level above high school or vocational training)	0.846	S	OR	Univariate
Li et al. (2010) [14]	Education level (Yi tribe)	1.06	S	OR	Multivariate
Zeng (2012) [16]	Education level	0.555	NS	OR	Multivariate
**2. Occupations**					
Yan et al. (2009) [12]	Intellectual job	0.268	S	OR	Univariate
Gao et al. (2013) [19]	Worker	1.78	S	OR	
	Housewife	0.49	S	OR	
**3. Economic factors**					
Zhang et al. (1990) [4]	Economic status	>2	S	OR	Univariate (p< .05 and/or OR >2)
	Family economic status	2.89	NR	OR	Multivariate
Wang et al. (2004) [10]	Income	0.51	S	OR	Multivariate

OR, odds ratio; S, significant; NS, not significant; NR, not reported.

(B) Lifestyle

Study Reference	Risk factor	Result	Significance	Statistics	Notes
**1. Addictions**					
s.n. (1986) [1]	Personal hobbies	-	NS	RR	
Dong et al. (1998) [6]	Cigarette smoking	1.76	NR	OR	
	Cigarette smoking	2	NR	OR	
Li et al. (2000) [7]	Smoking index (5 cigarette/day 3 number of smoking years) ≤200	2.2	NR	RR	Adjusted for age, IUD use, tubal ligation, no. of induced and

					spontaneous abortions, age at first birth, no. of births, months of breast feeding.
	Smoking index (5 cigarette/day 3 number of smoking years)200+	2.28	NR	RR	Same as above
	Smoking index (5 cigarette/day 3 number of smoking years) ≤200	1.61	NR	RR	Same as above
Kan et al. (2009) [12]	Smoking	2.213	S	OR	Univariate
Zhang et al. (2010) [13]	Smoking	1.92	S	OR	Multivariate
Li et al. (2010) [14]	Smoking (Yi tribe)	0.3	NS	OR	Multivariate
Li et al. (2011) [15]	Second hand smoking	1.844	S	OR	Multivariate
Zeng (2012) [16]	Smoking	4.888	S	OR	Multivariate
Liu et al. (2013) [17]	Smoking	3.177	S	OR	Multivariate
Jiang (2013) [18]	Smoking	3.552	S	OR	Multivariate
Nie et al. (2014) [21]	Second hand smoking <10 years	2.763	S	OR	Multivariate
	Second hand smoking 11-20 years	3.345	S	OR	Multivariate
	Second hand smoking <20 years	5.268	S	OR	Multivariate
**2. Dietary intake**					
Peng et al. (1991) [5]	Sichuan pickles	2.8	NR	OR	
Ma et al. (2005) [11]	Beans (g/d) <18.77	1	S	OR	Trend test analysis
	Beans (g/d) 18.77	0.49	S	OR	Same as above
	Beans (g/d) 31.92	0.26	S	OR	Same as above
	Beans (g/d) >56.51	0.52	S	OR	Same as above
	Dark vegetable (g/d) <167.11	1	S	OR	Same as above
	Dark vegetable (g/d) 167.11	0.56	S	OR	Same as above
	Dark vegetable (g/d) 198.58	0.41	S	OR	Same as above
	Dark vegetable (g/d) >253.35	0.25	S	OR	Same as above
	Fruit (g/d) 19.40	1	NS	OR	Same as above
	Fruit (g/d) <19.40	0.68	NS	OR	Same as above
	Fruit (g/d) <98.63	0.47	NS	OR	Same as above
	Fruit (g/d) >207.94	0.33	NS	OR	Same as above
	Meat (g/d) <22.19	1	S	OR	Same as above
	Meat (g/d) 22.19	0.72	S	OR	Same as above
	Meat (g/d) 46.30	0.48	S	OR	Same as above
	Liver (g/d) <50	1	NS	OR	Same as above
	Liver (g/d) ≥50	0.44	NS	OR	Same as above
	Beans (g/d) <18.77	1	NS	OR	Trend test analysis (Adjusted for age of menopause)
	Beans (g/d) 18.77	0.5	S	OR	Same as above
	Beans (g/d) 31.92	0.27	S	OR	Same as above
	Beans (g/d) >56.51	0.51	S	OR	Same as above
	Dark vegetable (g/d) <167.11	1	S	OR	Same as above
	Dark vegetable (g/d) 167.11	0.58	S	OR	Same as above
	Dark vegetable (g/d) 198.58	0.44	S	OR	Same as above
	Dark vegetable (g/d) >253.35	0.25	S	OR	Same as above
	Fruit (g/d) 19.40	1	NS	OR	Same as above

	Fruit (g/d) <19.40	0.68	NS	OR	Same as above
	Fruit (g/d) <98.63	0.49	NS	OR	Same as above
	Fruit (g/d) >207.94	0.31	NS	OR	Same as above
	Meat (g/d) <22.19	1	S	OR	Same as above
	Meat (g/d) 22.19	0.68	S	OR	Same as above
	Meat (g/d) 46.30	0.47	S	OR	Same as above
	Liver (g/d) <50	1	NS	OR	Same as above
	Liver (g/d) ≥50	0.43	NS	OR	Same as above
	Beans(g/d) <18.77	1	NS	OR	(trend test analysis, age of menopause, career, education level, average yearly income, smoking, frequency of shower, no. delivery, menopause age, age of first sexual intercourse adjustment)
	Beans(g/d) 18.77	0.7	NS	OR	Same as above
	Beans(g/d) 31.92	0.35	NS	OR	Same as above
	Beans(g/d) >56.51	0.71	NS	OR	Same as above
	Dark vegetable (g/d) <167.11	1	NS	OR	Same as above
	Dark vegetable (g/d) 167.11	0.63	NS	OR	Same as above
	Dark vegetable (g/d) 198.58	0.61	NS	OR	Same as above
	Dark vegetable (g/d) >253.35	0.34	NS	OR	Same as above
	Fruit (g/d) 19.40	1	NS	OR	Same as above
	Fruit (g/d) <19.40	0.91	NS	OR	Same as above
	Fruit (g/d) <98.63	0.43	NS	OR	Same as above
	Fruit (g/d) >207.94	0.89	NS	OR	Same as above
	Meat (g/d) <22.19	1	NS	OR	Same as above
	Meat (g/d) 22.19	0.86	NS	OR	Same as above
	Meat (g/d) 46.30	1.04	NS	OR	Same as above
	Liver (g/d) <50	1	NS	OR	Same as above
	Liver (g/d) ≥50	0.89	NS	OR	Same as above
Zeng (2012) [16]	Dietary	1.694	NS	OR	Multivariate
**3. General hygiene**					
Wang et al. (2004) [10]	Shower facility	0.317	S	OR	Multivariate
s.n. (1986) [1]	Infrequent washing of vulva	2.27	S	RR	
Zhang et al. (1989) [2]	Genital washing (daily)	1	NS	RR	
	Genital washing (not every day)	5.71	S	RR	
Zhang et al. (1989) [2]	Genital washing	>2	S	OR	And/or OR
Zhang et al. (1990) [4]	Rating of hygiene during menstruation cycle	2.61	NR	OR	
Zeng (2012) [16]	Personal habit	0.273	S	OR	Multivariate
**4. Hygiene: Use for sanitary napkin**					
s.n. (1986) [1]	Use of non-sanitary pads	-	NS	RR	
Zhang et al. (1989) [2]	No use of sanitary napkin	1	NS	RR	
	Use of sanitary napkin	0.28	S	RR	
Zhang et al. (1989) [2]	Use of sanitary pad	150.41	S	X2	
Zhang et al. (1990) [4]	Use of non-sanitary pads	>2	S	OR	And/or OR

Li et al. (2000) [7]	Material used during menstruation (sanitary paper)	1	NR	RR	Adjusted for age, IUD use, tubal ligation, no. of induced/spontaneous abortions, age at first delivery, no. of births, and months of breast feeding.
	Material used during menstruation (non-sanitary materials)	1.73	NR	RR	Same as above
	Material used during menstruation (sanitary paper)	1	NR	RR	Same as above
	Material used during menstruation (non-sanitary materials)	1.35	NR	RR	Same as above
**5. Hygiene: Sexual habits**					
Li et al. (2010) [14]	Washing after sex (Han tribe)	1.79	NS	OR	Multivariate
Li et al. (2011) [15]	Hygiene during sexual activity	0.329	S	OR	Multivariate
**6. Living conditions**					
s.n. (1986) [1]	Living condition	-	NS	RR	
7. Tea intake					
Li et al. (2011) [15]	Frequent consumption of tea	0.558	S	OR	Multivariate
Nie et al. (2014) [21]	Frequent consumption of tea	0.233	S	OR	Multivariate

OR, odds ratio; RR, relative risk; S, significant; NS, not significant; IUD, intrauterine device; s.n., sine nomine.; NR, not reported.

(C) Sexual behaviour and marital status

Study Reference	Risk factor	Result	Significance	Statistics	Notes
**1. Age at sexual debut**					
Zhang et al. (1989) [2]	Age at first sexual intercourse <16	1	NS	RR	
	Age at first sexual intercourse 17-18	0.78	NS	RR	
	Age at first sexual intercourse 19-20	0.74	NS	RR	
	Age at first sexual intercourse >21	0.35	NS	RR	
Cai et al. (2008) [8]	Age at first intercourse >23	-	NR	OR	Multivariate
	Age at first intercourse 21-23	0.41	NR	OR	Multivariate
	Age at first intercourse 18-20	1.46	NS	OR	Multivariate
	Age at first intercourse <18	3.71	S	OR	Multivariate
Wang et al. (2004) [10]	Age of first sexual contact ≤20	3.99	S	OR	Multivariate
Yan et al. (2009) [12]	Age at first sexual intercourse <20	4.163	S	OR	Univariate
Li et al. (2010) [14]	First sex age (Yi people)	2.09	NS	OR	Multivariate
Zeng (2012) [16]	Age at sexual debut (age not specified)	0.436	S	OR	Multivariate
Liu et al. (2013) [17]	Age at first intercourse ≤20	4.314	S	OR	Multivariate
Gao et al. (2013) [19]	Sex for first time ≥21 years	2.21	S	OR	
Wang and Zhou (2014) [20]	Age at first sexual intercourse	0.521	S	OR	Multivariate
**2. Age at marriage**					

Zhang et al. (1989) [2]	Age at marriage <16	1	NS	RR	
	Age at marriage 17-18	0.92	NS	RR	
	Age at marriage 19-20	0.8	NS	RR	
	Age at marriage >21	0.38	NS	RR	
Zhang et al. (1990) [4]	Age at first marriage for cases with ≥2 marriages	1.47	NR	OR	
Peng et al. (1991) [5]	Age at first marriage	0.166	NS	OR	
Dong et al. (1998) [6]	Age at marriage >26	1	NR	OR	
	Age at marriage NEVER	0.3	NR	OR	
	Age at marriage <19	0.1	NR	OR	
	Age at marriage 20-25	0.5	NR	OR	
	Age at marriage >26	1	NR	OR	
	Age at marriage NEVER	0.34	NR	OR	
	Age at marriage <19	0.11	NR	OR	
	Age at marriage 20-25	0.65	NR	OR	
Li et al. (2000) [7]	Age when first married (years) 26+	1	NR	RR	Adjusted for age, IUD use, tubal ligation, no. of induced and spontaneous abortions, age at first birth, no. of births, months of breast feeding.
	Age when first married (years) 23-25	1.85	NR	RR	
	Age when first married (years) 20-22	3.63	NR	RR	
	Age when first married (years) 13-19	5.77	NR	RR	
Wang et al. (1992) [9]	Marriage age	0.598	S	Regres. coeff.	Multivariate
Wang et al. (2004) [10]	Marriage age ≤20	16.07	NR	OR	
	Marriage age 21	4.18	S	OR	
Nie et al. (2014) [21]	Marriage age	0.521	S	OR	Multivariate
3. Number of sexual partners					
s.n. (1986) [1]	Complicated sexual history	2.11	S	RR	
Zhang et al. (1989) [2]	No. of non-marital sexual partners (1)	1	S	RR	
	No. of non-marital sexual partners (2)	2.21	S	RR	
	No. of non-marital sexual partners (>2)	6.68	S	RR	
	No. of non-marital sexual partners none + No adjustment for screening history	1	S	RR	
	No. of non-marital sexual partners 1 + No adjustment for screening history	2.33	S	RR	
	No. of non-marital sexual partners 2 or more + No adjustment for screening history	4.23	S	RR	
	No. of non-marital sexual partners none + adjustment for screening history	1	S	RR	

	No. of non-marital sexual partners 1 + adjustment for screening history	2.47	S	RR	
	No. of non-marital sexual partners >2 + adjustment for screening history	3.87	S	RR	
Zhang et al. (1990) [4]	Complicated sexual history	2.53	NR	OR	
Cai et al. (2008) [8]	Lifetime no. of sexual partners 0-1	-	NR	OR	Multivariate
	Lifetime no. of sexual partners >2	1.01	NS	OR	Multivariate
Kan et al. (2009) [12]	Sexual partner >3	4.506	S	OR	Univariate
Zeng (2012) [16]	No. of partners <1	7.089	S	OR	Multivariate
Gao et al. (2013) [19]	Sex partner >1	1.92	S	OR	
**4. Number of marriages**					
s.n. (1986) [1]	Married twice	1.45	S	RR	
	Married three times	1.91	S	RR	
Peng et al. (1991) [5]	No. of marriages	0.9	NR	OR	
Wang et al. (1991) [9]	Marriage times	1.609	S	Regres. coeff.	Univariate
5. Number of sexual partner of partner					
s.n.(1986) [1]	Complicated sexual history for husband	2.65	S	RR	
Zhang et al. (1989) [2]	No. of husband's non-marital sexual partners none + no adjustment for screening history	1	S	RR	
	No. of husband's non-marital sexual partners 1 + no adjustment for screening history	0.78	S	RR	
	No. of husband's non-marital sexual partners 2 or more + no adjustment for screening history	4.55	S	RR	
	No. of husband's non-marital sexual partners none + adjusted for screening history	1	S	RR	
	No. of husband's non-marital sexual partners 1 + adjusted for screening history	0.93	S	RR	
	No. of husband's non-marital sexual partners >2 adjusted for screening history	5.9	S	RR	
	No. of husband's other sexual partners none	1	S	RR	
	No. of husband's other sexual partners 1	1.8	S	RR	
	No. of husband's other sexual partners >2	4.69	S	RR	
**6. Duration of marriage**					
Dong et al. (1998) [6]	Married years <4	1	NR	OR	
	Married years 5-9	6.1	NR	OR	
	Married years 10-19	6.5	NR	OR	

	Married years >20	NA	NR	OR	
	Married years <4	1	NR	OR	
	Married years 5-9	5.9	NR	OR	
	Married years 10-19	3.1	NR	OR	
Gao et al. (2013) [19]	Sex partner lasting >10 year	1.81	S	OR	
**7. Bleeding sex**					
Li et al. (2010) [14]	Bleeding during sex (Yi tribe)	6.79	S	OR	Multivariate
	Bleeding during sex (Han tribe)	2.95	NS	OR	Multivariate
**8. Marriage status**					
Zhang et al. (2010) [13]	Marital status	1.36	S	OR	Multivariate
**9. Sex before marriage**					
Wang et al. (1992) [9]	Sex before marriage	1.386	NS	Regres. coeff.	Univariate
**10.Sex since delivery/during menstrual cycles**					
s.n. (1986) [1]	Sex during menstrual cycles	3	NS	RR	
	Sex since delivery	1.37	NS	RR	
**11. Number of marriage partners**					
Zhang et al. (1989) [2]	No. of husband's marriages	1	NS	RR	
	No. of husband's marriages 2	1.48	NS	RR	
	No. of husband's marriages 3	0.85	NS	RR	
Peng et al. (1991) [5]	No. of marriages	0.9	NR	OR	

OR, odds ratio; RR, relative risk; S, significant; NS, not significant; NR, not reported; NA, not available; Regres.coeff., regression coefficient; IUD, intrauterine device;

(D) Gestational risk factors

Study Reference	Risk factor	Result	Significance	Statistics	Notes
**1. Contraception**					
Li et al. (2000) [7]	Tubal ligation	1.08	NR	OR	Univariate; Adjusted for age, years of school, age when first married, smoking index, sexual intercourse during menstruation, unsanitary materials used during menstrual periods, no. of births, and frequency of Pap smears.
	Age at tubal ligation (years ≤32)	1	NR	OR	
	Age at tubal ligation (years ≥33)	0.54	NR	OR	
	No. of years since tubal ligation (none)	1	NR	OR	
	No. of years since tubal ligation (1-10)	0.63	NR	OR	
	No. of years since tubal ligation (≥11)	0.9	NR	OR	
	Years since sterilization 1-10 (Age ≤32)	0.31	NR	RR	
	Years since sterilization 1-10 (Age 33+)	0.63	NR	RR	

	Years since sterilization 11+ (Age ≤32)	0.94	NR	RR	
	Years since sterilization 11+ (Age 33+)	1.24	NR	RR	
	IUD use	0.89	NR	RR	
	Age at first IUD use ≤32	0.51	NR	RR	
	Age at first IUD use 33+	0.76	NR	RR	
	Number of years of IUD use 1-10	0.63	NR	RR	
	Number of years of IUD use 10+	0.68	NR	RR	
	Age at first IUD use (≤32 yrs) + Years of IUD use 1-10	0.63	NR	RR	
	Age at first IUD use (≤32 yrs) + Years of IUD use 11+	0.44	NR	RR	
	Age at first IUD use (33+yrs) + Years of IUD use 1-11	0.69	NR	RR	
	Age at first IUD use (33+ yrs) + Years of IUD use 11+	0.9	NR	RR	
Wang et al. (1992) [9]	Contraception	0.2877	NS	Regres. coeff.	Univariate
Kan et al. (2009) [12]	Condom use	0.653	S	OR	Univariate
Zhang et al. (2010) [13]	Contraception measures	0.44	S	OR	Multivariate
Li et al. (2010) [14]	Contraception (Han tribe)	0.57	NS	OR	Multivariate
Zeng (2012) [16]	Oral contraception	2.419	S	OR	Multivariate
Liu et al. (2013) [17]	Condom use	0.447	S	OR	Multivariate
Gao et al. (2013) [19]	Married times >1	6.92	S	OR	
	Without contraception	3.63	S	OR	
	Oral contraception	0.35	S	OR	
	With condom	0.18	S	OR	
	Other methods for contraception	5.71	S	OR	
	With contraception currently	0.63	S	OR	
	With condom currently	0.08	S	OR	
**2. Number of pregnancies**					
Dong et al. (1998) [6]	Pregnancy never	1	NR	OR	
	Pregnancy 1-2 times	0.47	NR	OR	
	Pregnancy >3 times	0.86	NR	OR	
	Parity never	1	NR	OR	
	Parity 1-2 times	1.1	NR	OR	
	Parity >3 times	1.9	NR	OR	
	Pregnancy never	1	NR	OR	
	Pregnancy 1-2 times	0.24	NR	OR	
	Pregnancy >3 times	0.72	NR	OR	
	Parity never	1	NR	OR	
	Parity 1-2 times	1.2	NR	OR	
	Parity >3 times	1.5	NR	OR	
Cai et al. (2008) [8]	No of pregnancies 0-1	-	NR	OR	Multivariate
	No. of pregnancies 2-3	1.67	NS	OR	Multivariate
	No. of pregnancies >4	3.49	NS	OR	Multivariate

Wang et al. (1992) [9]	No. of pregnancies	0.0354	NS	Regres. coeff.	Univariate
Wang et al. (2004) [10]	No. of pregnancies>4	1.47	S	OR	
	No. of pregnancies	0.0354	NS	Regres. coeff.	Univariate
Kan et al. (2009) [12]	No. of pregnancies <3	1.371	S	OR	Univariate
Li et al. (2010) [14]	No. of pregnancies (Yi tribe)	4.16	S	OR	Multivariate
	No. of pregnancies (Han tribe)	1.83	NS	OR	Multivariate
Zeng (2012) [16]	No. of pregnancies	1.447	NS	OR	Multivariate
Wang and Zhou (2014) [20]	No. of pregnancies	3.776	S	OR	Multivariate
**3. History of non-full-term live birth termination**					
s.n. (1986) [1]	Miscarriage, still birth, difficult labour	-	NS	RR	
Wang et al. (1992) [9]	Natural abortion	0.0554	NS	Regres. coeff.	Univariate
Yan et al. (2009) [12]	No. of non-full-term live birth/termination >3	3.946	S	OR	Univariate
Zeng (2012) [16]	Artificial abortion	3.91	S	OR	Multivariate
Liu et al. (2013) [17]	Incidence of abortion ≥4	2.451	S	OR	Multivariate
Wang and Zhou (2014) [20]	No. of non-full-term live birth/termination	6.116	S	OR	Multivariate
**4. Number of deliveries**					
Wang et al. (1992) [9]	No. of delivery	0.0657	NS	Regres. coeff.	Univariate
Kan et al. (2009) [12]	No. of delivery >3	2.795	S	OR	Univariate
Li et al. (2010) [14]	No. of delivery (Yi people)	1.94	S	OR	Multivariate
	No. of delivery (Han people)	2.18	S	OR	Multivariate
Li et al. (2011) [15]	No. delivery >2	2.267	S	OR	Multivariate
Zeng (2012) [16]	No. of delivery	0.436	NS	OR	Multivariate
Nie et al. (2014) [21]	No. of delivery	1.393	S	OR	Multivariate
**5. Age at first delivery**					
Wang et al. (1992) [9]	First delivery age	0.539	S	Regres. coeff.	Univariate
Wang et al. (2004) [10]	First pregnancy age ≤20	13.71	NR	OR	
	First pregnancy age 21	3.23	S	OR	
Kan et al. (2009) [12]	Age at first delivery <21 years old	1.295	S	OR	Univariate
Zeng (2012) [16]	First delivery age	0.279	S	OR	Multivariate
Li et al. (2010) [14]	First delivery age (Yi tribe)	1.4	NS	OR	Multivariate
**6. Age at first pregnancy**					
Wang et al. (2004) [10]	First pregnancy age ≤20	13.71	NR	OR	
	First pregnancy age 21	3.23	S	OR	
Kan et al. (2009) [12]	Age at first pregnancy <21 years old	2.283	S	OR	Univariate
Liu et al. (2013) [17]	Age at first pregnancy ≤20	2.432	S	OR	Univariate
Gao et al. (2013) [19]	Pregnancy age for first time ≤23 years old	1.74	S	OR	

**7. Age at first menstruation**					
s.n. (1986) [1]	Age at first menstruation	-	NS	RR	
Wang et al. (1992) [9]	First menstrual cycle age	-0.047	NS	Regres. coeff.	Univariate
Wang and Zhou (2014) [20]	Age at first menstruation (<14 years old)	3.242	S	OR	Multivariate
**8. Number of live births**					
Cai et al. (2008) [8]	No. of live births 0-1	-	NR	OR	Multivariate
	No. of live births 2	6.05	NS	OR	Multivariate
	No. of live births 3	9.06	NS	OR	Multivariate
	No. of live births 3+	16.82	S	OR	Multivariate
Wang et al. (2004) [10]	No. births	2.29	S	OR	Multivariate
Liu et al. (2013) [17]	No. of births ≥4	2.375	S	OR	Multivariate
**9. Age at last menstruation**					
s.n. (1986) [1]	Age at menopause	-	NS	RR	
Wang et al.(1992) [9]	Menopause age	0.0593	NS	Regres. coeff.	Univariate
Wang et al. (2004) [10]	Menopause age	0.68	S	OR	Multivariate
Kan et al. (2009) [12]	Menopause	0.526	S	OR	Univariate
Gao et al. (2013) [19]	Menopause age	0.54	S	OR	
**10. Time between delivery/live birth**					
Wang et al. (1992) [9]	Time between delivery/live births/pregnancies	0.1026	NS	Regres. coeff.	Univariate
**11. Interval of menstruation**					
Zeng (2012) [16]	Menstrual period cycle	0.069	S	OR	Multivariate

IUD, intrauterine device; OR, odds ratio; RR, relative risk; S, significant; NS, not significant; NR, not reported; Regres.coeff., regression coefficient.

(E) Cervical screening and other diseases

Study Reference	Risk factor	Result	Significance	Statistics	Notes
**1.Gynaecological disorder/disease**					
s.n. (1986) [1]	Cervical erosion 1	5	S	RR	
	Cervical erosion 2	3.89	S	RR	
	Other gynaecological disease history	1.54	NS	RR	
	Family history of gynaecological disease	1.44	NS	RR	
Zhang et al. (1990) [4]	Cervical erosion	3.01	NR	OR	
Wang et al. (1992) [9]	Cervical erosion	1.099	S	Regres. coeff.	Univariate
	Cervical erosion	1.963	S	Regres. coeff.	Univariate
	Cervical cancer history in family	2.773	S	Regres. coeff.	Univariate
	Cervical cancer history in family	3.426	S	Regres. coeff.	Univariate

	Tumour history in family	0.6061	NS	Regres. coeff.	Univariate
Wang et al. (2004) [10]	Disease history	2.887	S	OR	Multivariate
Yan et al. (2009) [12]	Disease history	1.447	S	OR	Univariate
Zhang et al. (2010) [13]	Trichomonas vaginitis	2.37	S	OR	Multivariate
	Family history of cervical cancer	4.98	S	OR	Multivariate
	Family history of endometrial cancer	4.52	S	OR	Multivariate
Zeng (2012) [16]	Gynaecological history	2.833	S	OR	Multivariate
	Family cancer history	1.677	NS	OR	Multivariate
Liu et al. (2013) [17]	Family history of cancer	1.313	S	OR	Univariate
Jiang (2013) [18]	Pelvic inflammation	2.377	S	OR	Multivariate
Wang and Zhou (2014) [20]	Cervical inflammation	5.496	S	OR	Multivariate
**2. HPV infection**					
Cai et al. (2008) [8]	HPV positive	75.79	S	OR	Multivariate
Yan et al. (2009) [12]	HPV-16 positive	32.256	S	OR	Univariate
	HPV-52 positive	6.163	S	OR	Univariate
Liu et al. (2013) [17]	HPV infection	20.971	S	OR	Multivariate
Jiang (2013) [18]	HPV infection	8.743	S	OR	Multivariate
Gao et al. (2013) [19]	HPV positive	108.75	S	OR	
Wang and Zhou (2014) [20]	HPV infection	25.312	S	OR	Multivariate
**3. Mental health**					
s.n. (1986) [1]	Mental health influence	-	NS	RR	
Wang et al. (1992) [9]	Mentally abused record	1.386	S	Regres. coeff.	Univariate
Yan et al. (2009) [12]	Mental health history	1.468	S	OR	Univariate
Jiang (2013) [18]	Work pressure	4.738	S	OR	Multivariate
**4. Cervical cancer screening history**					
Zhang et al. (1989) [3]	Years since last negative smear 0-2	1	NS	RR	
	Years since last negative smear >4	6.3	NS	RR	
	No. of negative smears 0-1	1	NS	RR	
	No. of negative smears 2-3	0.8	NS	RR	
	No. of negative smears >3	0.42	NS	RR	
Cai et al. (2008) [8]	Interval since last Pap smear never	1		OR	Multivariate
	Interval since last Pap smear never ≥5 years	4.75	NS	OR	Multivariate
	Interval since last Pap smear never <5 years	9.52	S	OR	Multivariate
Li et al. (2010) [14]	Regular gynaecological checking (Yi tribe)	1.13	NS	OR	Multivariate
	Regular gynaecological check-up (Han tribe)	1.21	NS	OR	Multivariate
	Gynaecological check-up location (Han tribe)	2.76	NS	OR	Multivariate
**5. Sexual Transmitted Disease**					

Jiang (2013) [18]	STD	7.561	S	OR	Multivariate

OR, odds ratio; RR, relative risk; S, significant; NS, not significant; NR, not reported; Regres.coeff., regression coefficient; HPV, human papillomavirus; STD, sexually transmitted disease.

(F) Other factors

Study Reference	Risk factor	Result	Significance	Statistics	Notes
**1. Foreskin (e.g. phimosis, circumcision)**					
s.n. (1986) [1]	Husband with phimosis	2.4	NR	RR	
Zhang et al. (1990) [4]	Partner with phimosis	>2	S	OR	And/or OR
Kan et al. (2009) [12]	Partner with phimosis	1.921	S	OR	Univariate
Liu et al. (2013) [17]	Spouse has phimosis	2.138	S	OR	Multivariate
	Spouse has circumcision	0.513	S	OR	Multivariate
**2. Biomarker levels**					
Ma et al. (2005) [11]	Retinoic acid (μgRE/ dL) 170.10	1	S	OR	Trend analysis
	Retinoic acid (μgRE/dL) 311.16	0.52	S	OR	Same as above
	Retinoic acid (μgRE/dL) 508.21	0.42	S	OR	Same as above
	Retinoic acid (μgRE/dL) 796.01	0.36	S	OR	Same as above
	Folic acid (μg/dL) <210.18	0.4	S	OR	Same as above
	Folic acid (μg/dL) 210.18	1	S	OR	Same as above
	Folic acid (μg/dL) 248.65	0.82	S	OR	Same as above
	Folic acid (μg/dL) 291.78	0.67	S	OR	Same as above
	Folic acid (μg/dL) 365.54	0.41	S	OR	Same as above
	Folic acid (μg/dL) >365.54	0.33	S	OR	Same as above
	Retinoic acid (μgRE/dL) 170.10	1	S	OR	Same as above
	Retinoic acid (μgRE/dL) 311.16	0.49	S	OR	Same as above
	Retinoic acid (μgRE/dL) 508.21	0.38	S	OR	Same as above
	Retinoic acid (μgRE/dL) 796.01	0.35	S	OR	Same as above
	Folic acid (μg/dL) <210.18	0.41	S	OR	Same as above
	Folic acid (μg/dL) 210.18	1	NS	OR	Same as above
	Folic acid (μg/dL) 248.65	0.74	NS	OR	Same as above
	Folic acid (μg/dL) 291.78	0.6	NS	OR	Same as above
	Folic acid (μg/dL) 365.54	0.17	NS	OR	Same as above
	Folic acid (μg/dL)>365.54	0.29	NS	OR	Same as above
	Retinoic acid (μgRE/dL) 170.10	1	NS	OR	Adjusted by age, occupation, educational level, income per capita, smoking, bathing frequency, parity, menopause and the first sexual intercourse age
	Retinoic acid (μgRE/dL) 311.16	0.67	NS	OR	Same as above
	Retinoic acid (μgRE/dL) 508.21	0.73	NS	OR	Same as above
	Retinoic acid (μgRE/dL) 796.01	0.68	NS	OR	Same as above
	Folic acid (μg/dL) <210.18	0.97	NS	OR	Same as above
	Folic acid (μg/dL) 210.18	1	NS	OR	Same as above
	Folic acid (μg/dL) 248.65	0.9	NS	OR	Same as above
	Folic acid (μg/dL) 291.78	0.79	NS	OR	Same as above

	Folic acid (µg/dL) 365.54	0.43	NS	OR	Same as above
	Folic acid (µg/dL) >365.54	0.36	NS	OR	Same as above
Li et al. (2011) [15]	Folic acid level (10-15 vs. <10)	0.472	S	OR	Multivariate
	Folic acid level (>15 vs. <10)	0.389	S	OR	Multivariate
Nie et al. (2014) [21]	Folic acid in the blood 11.46-15.76	0.57	S	OR	
	Folic acid in the blood 15.76-19.93	0.462	S	OR	
	Folic acid in the blood ≥19.93	0.477	S	OR	
**3. Other bodily measure**					
Zeng (2012) [16]	BMI	1.585	NS	OR	Multivariate
	Waist to hip ratio	4.193	S	OR	Multivariate
	Blood pressure	2.132	NS	OR	Multivariate
Gao et al. (2013) [19]	Systolic pressure >90mm Hg	0.12	S	OR	
**4. Knowledge**					
Li et al. (2010) [14]	Understanding own cervix (Han tribe)	0.08	S	OR	Multivariate
Zeng (2012) [16]	Knowledge of CC	0.804	NS	OR	Multivariate
**5. Penile disease/cancer of the husband**					
Kan et al. (2009) [12]	Partner with penile diseases	3.475	S	OR	Univariate
Liu et al. (2013) [17]	Spouse has penile cancer	1.207	S	OR	Univariate
**6. Frequently visit hospital**					
Zhang et al. (1990) [4]	Frequent attendance to hospital	>2	S	OR	And/or OR

OR, odds ratio; RR, relative risk; S, significant; NS, not significant; NR, not reported; CC, cervical cancer; BMI, body mass index; s.n., sine nome

Supplementary Table 6: Counts of risk factors under different categorical ranges of odds ratios

	Number of studies						
	OR=>10	OR =5-10	OR =1-5	OR=1	OR<1	Not significant	Significance not reported
**Risk factor 1: Socio-demographics**							
Education			1	1		1	
Occupation				1	2		
Economic status			1		1		1
**Risk factor 2: Lifestyle**							
Addictions		1	8			1	5
Dietary intake				5	16	31	1
General hygiene		1	2		2	1	1
Hygiene: use for sanitary napkin	1		1		1	2	4
Hygiene-sexual habits					1	1	
Living conditions						1	
Tea intake					2		
**Risk factor 3: sexual behaviour and marital status**							
Age at sexual debut			5		2	6	2
Age at marriage			1		2	5	14
No. of sexual partners of partners/spouse		2	8	3		1	2
No. of marriages			3				1
No. of sexual partner of partner		1	4	3	2		
Duration of marriages			1				7
Bleeding during sex		1				1	
Marriage status			1				
Sex before marriage						1	
Frequency of sex since delivery/during menstrual cycle						2	
No. of marriages of partners						3	1
**Risk factor 4: gestational risk factors**							
Contraception		2	2		7	2	19
No. of pregnancies			4			6	13
History of non-full-term delivery birth/termination		1	3			2	
No. of deliveries			5			2	
Age of first delivery			2		2	1	1
Age at first pregnancy			4				1
Age at first menstruation			1			2	
No. of live births	1		2			2	1
Age at last menstruation					3	2	
Time between deliveries/live birth/pregnancies						1	
Interval of menstruation					1		
**Risk factor 5: cervical cancer screening and gynaecological diseases**							
Gynaecological disorders		1	14			4	1
HPV infections	5	2					
Mental health			3			1	
Cervical screening history		1				9	
Sexually transmitted disease		1					

**Risk factor 6: other factors**							
Foreskin status			3		1		1
Biomarker level				3	17	15	
Other bodily measures			1		1	2	
Knowledge (i.e. sex, cervix)					1	1	
Penile disease/cancer of husband				2			
Frequency of hospital visits			1				

NO. number

OR, odds ratio

References

1. s.n. Epidemiologic factors in cervical cancer--investigation on 306 pairs of partners. Jiangxi Co-operative Group of Cervical Cancer. *China Cancer*. 1986; 8: 444-6.
2. Zhang Z, Parkin D, Yu S, Estève J and Yang X. Risk factors for cancer of the cervix in a rural Chinese population. *Int J Cancer*. 1989; 43: 762-7.
3. Zhang Z, Parkin D, Yu S, Esteve J, Yang X and Day N. Cervical screening attendance and its effectiveness in a rural population in China. *Cancer Detect Prev*. 1989; 13: 337-42.
4. Zhang G and Xu A. Conditional logistic regression analysis and path analysis of risk factors of cervical cancer. *Chinese Journal of Epidemiology*. 1990; 11: 212-6.
5. Peng H, Liu S, Mann V, Rohan T and Rawls W. Human papillomavirus types 16 and 33, herpes simplex virus type 2 and other risk factors for cervical cancer in Sichuan Province, China. *Int J Cancer*. 1991; 47: 711-6.
6. Dong Y, Sasagawa T, Fang S, et al. Human papillomavirus,Chlamydia trachomatis, and other risk factors associated with cervical cancer in China. *Int J Clin Oncol*. 1998; 3: 81-7.
7. Li H, Thomas D, Jin S and Wu F. Tubal sterilization and use of an IUD and risk of cervical cancer. *J Womens Health Gend Based Med*. 2000; 9: 303-10.
8. Cai H, Ding X, Zhou Y and Lie D. Risk factors for cervical cancer in China: a case-control study. *Eur J Gynaecol Oncol*. 2008; 29: 72-5.
9. Wang Z, Chen Z and Wen H. Multivariate Analysis of Cervical Cancer Risk Factors (100 Case - Control Study). *China Cancer*. 1992; 10: 340-2.
10. Wang J, Gao E, Cheng Y, Yan J and Ding L. Case-control study on risk factors of cervical cancer. *Chinese Journal of Public Health*. 2004; 20: 161-2.
11. Ma X, Wang J, Cheng Y, Yan J and Zhou L. Case-control study: the relationship between dietary factors and cervical cancer. *Chinese Journal of Public Health*. 2005; 21: 312-4.
12. Kan S, Lu X, Wang C, Zheng GX, Li W and Zhang X. Case-control Study on Risk Factors of Cervical Cancer in Shandong Province. *J Shandong Univ (Health Sci)*. 2009; 47: 122-4.
13. Zhang S, Zhao Q, Wang T and Deng X. Analysis of Cervical Cancer Risk Factor in Beijing 1: 3 Case-Control Epidemiological Survey. *Maternal and Child Health Care China*. 2010; 25: 947-9.
14. Li Y, Gong X, Ma Y, Luo L and Yang W. Epidemiological Study on Risk Factors of Cervical Cancer among Han and Yi Women in Liangshan *Modern Journal of Integrated Traditional Chinese and Western Medicine*. 2010; 19: 4535-8.
15. Li J, Fan Y, Xu R and ZHOU Y. Case-control Study of Cervical Cancer Risk Factors. *Chinese Journal of Public Health*. 2011; 27: 264-5.
16. Zeng X. Case-control study on risk factors of cervical cancer. *China Modern Medicine*. 2012; 19: 169-70.
17. Liu S, Yang Y and Yang H. Cervical Cancer Risk Factor in Wudu, Gansu Province. *Journal of Modern Oncology*. 2013; 21: 2793-5.
18. Jiang B. Analysis of risk factors related to cervical cancer and precancerous lesions. *Chinese and foreign medical research*. 2013; 11: 30-1.
19. Gao Y, Yao W and Wang S. Case-control Study on the Risk Factors of Cervical Cancer in Shenyang City. *Journal of Practical Oncology*. 2013; 27: 130-2.
20. Wang J and Zhou C. Epidemiological study and analysis of cervical cancer risk factors. *Chinese and foreign medical research*. 2014; 12: 79-80.
21. Nie X, Xu R and Li J. Effect of folic acid and risk factors on cervical cancer. *Maternal and Child Health Care China*. 2014; 29: 106-9.
